# A potential role of microRNAs in protein accumulation in cellular senescence analyzed by bioinformatics

**DOI:** 10.1371/journal.pone.0179034

**Published:** 2017-06-07

**Authors:** Yuequan Zhu, Kai Xiong, Jiangcheng Shi, Qinghua Cui, Lixiang Xue

**Affiliations:** 1 Medical Research Center, Department of Radiation Oncology, Peking University Third Hospital, Beijing, China; 2 Department of Biomedical Informatics, School of Basic Medical Sciences, Peking University, Beijing, China; Huazhong University of Science and Technology, CHINA

## Abstract

Cellular senescence is an important protective mechanism against cell proliferation and has critical roles in aging and aging-related disease. Recently, one interesting observation is that the protein abundance is higher in senescent cells than that in young cells. So far, some factors were presented to interpret this observation, such as active protein synthesis linked with autophagy, mTOR, and oxidative stress. Here, applying bioinformatic analysis of microRNA profiles in young cells and aging cells, we revealed that globally senescent cells show lower miRNA abundance than that in young cells, suggesting that the repression of protein synthesis by miRNA in senescent cells could be largely attenuated. This finding provides clues that protein accumulation in cellular senescence could be associated with lower miRNA abundance in aging cells.

## Introduction

Cellular senescence is the process of accumulative changes in both cellular structure and function that disrupts metabolism, resulting in deterioration and irreversible cell cycle arrest. Cells undergo senesce normally after a certain times divisions in vitro and can also be induced to senesce by toxins, irradiation, and oncogenes, etc. [1]. Cellular senescence represents a robust and important tumor-suppressive barrier that inhibits cell proliferation and malignancy[2]. Because cells are the basic blocks of organisms, cellular senescence is account for the whole aging process. So far, it has been well documented that senescence plays critical roles in aging and aging-related disease, such as cancer[2], cardiovascular disease[3], and diabetes[4]. Therefore, cellular senescence is becoming one important mechanism for understanding a number of physiological and pathological processes.

Recently, one amazing observation is that the total amount of proteins in senescent cells increased compared with that in young cells, suggesting that protein synthesis seems to be activated in senescent cells [5–7]. So far, a number of studies on the mechanism were presented to explain the above phenomenon. For example, oxidative stress resulted from aging may result in the accumulation of damaged proteins[5]. Although how protein synthesis and autophagy are activated in senescence remains largely unknown, it is accepted that oncogene, such as Ras could activate autophagy, which further facilitates protein synthesis and results in the cellular senescence [6]. These mechanisms greatly improved our understanding to cellular senescence; however, more evidences are needed to provide a comprehensive understanding of senescence. Given that protein-coding genes are negatively regulated by miRNAs, it remains unknown whether miRNAs accumulated or decreased in aging cells. Here, by analyzing miRNA expression profiles of senescent cells and young cells, we raised our hypothesis that repressed miRNA expression might partly explain the accumulated proteins in senescent cells.

miRNAs mainly function as negative gene regulators to repress mRNA translation or degrade mRNAs through binding the 3’UTR of targets [8]. As one class of small noncoding RNAs, miRNA plays critical roles in many biological processes. It has been revealed that miRNA-related dysfunctions are associated with a wide spectrum of diseases, including aging and aging-related disease[9]. Recently, emerging studies have also shown that miRNAs are involved in the regulation during cellular senescence as well [10, 11]. Thus, miRNAs might serve as a new indicating molecules during senescence transition and may shed light on the understanding cellular senescence and aging. However, crucial questions need to be urgently addressed: Is miRNA synthesis also activated like protein synthesis in senescent cells? Do miRNAs contribute to the accumulated proteins in senescent cells?

Here by an integrative analysis of miRNA profiles in young and aging cells, we revealed that globally senescent cells show lower miRNA abundance than young cells, suggesting that the repression on protein synthesis by miRNAs in senescent cells could be largely released. This finding suggests that lower miRNA abundance in aging cells could be another factor in interpreting the phenomenon of protein accumulation in cellular senescence.

## Materials and methods

### miRNA expression datasets

We obtained the miRNA expression profiles of young and replicative senescent human umbilical vein endothelial cells from the GEO database (GEO accession number: GSE37092). We obtained the miRNA expression profiles of young and oncogenic H-Ras V12-induced senescent (RIS) IMR90 cells from the CuiLab (SupplData at http://www.cuilab.cn/), which identified miRNA profiles across more than ten types of human cells for the systems study of miRNA stability. In addition to test the difference between young and old cells, we also obtained the data from the organisms. The miRNA expression profiles in young (developmental) and aging macaque brain from the GEO database (GEO dataset: GSE18013) were applied to conduct the analysis. To solid this study, we accessed two more datasets, the miRNA expression data in human aging adipose and the umbilical vein endothelial cells from the GEO database (GEO accession number: GSE64566 and GSE37092). These datasets were summarized in Table 1.

**Table 1:** List of the miRNA expression datasets used in this study.

No.	miRNA expression datasets of	source
1	Young and replicative senescent human umbilical vein endothelial cells	GEO: GSE37092
2	Young and oncogenic H-Ras V12-induced senescent (RIS) IMR90 cells	http://www.cuilab.cn
3	Young (developmental) and aging macaque brain	GEO: GSE18013
4	Human aging adipose	GEO: GSE64566
5	The umbilical vein endothelial cells	GEO: GSE37092

### Microarray analysis

For the datasets from the public database, we directly downloaded the normalized data. For the log2 transformed datasets, we calculated the value before log2 transformation. For the data produced in our lab, we used GenePix Pro 6.0 software (Axon) for grid alignment and data extraction of the scanned images. Then, we removed the miRNAs that Foreground-Background intensities are smaller than 30, which were considered to be not reliable. Finally, we normalized the miRNA expression data based on the expression level of U6. It should be noted that because these datasets are from different platforms and labs, they could be normalized using different methods. So they could be not in the same scale. But this does not affect the comparison of the aging and young samples within one dataset. The comparison of miRNA expression levels between aging and young cells is performed using the Wilcoxon test, which is a nonparametric test that compares two paired groups and used to decide whether the two population distributions (median values) are identical.

### Oncogenic analysis of the miRNAs upregulated/downregulated in aging cells or tissues

TAM[12] software, a miRNA set enrichment analysis tool was used to analyze the oncogenic characteristics of the deregulated miRNAs from the three miRNA profile datasets. miRNA set was defined as a group of miRNAs which show the same or similar characteristics. Using TAM, we can infer the patterns and rules behind a list of miRNAs.

## Results

### miRNA expression level in young and aging cells

**Fig 1:**
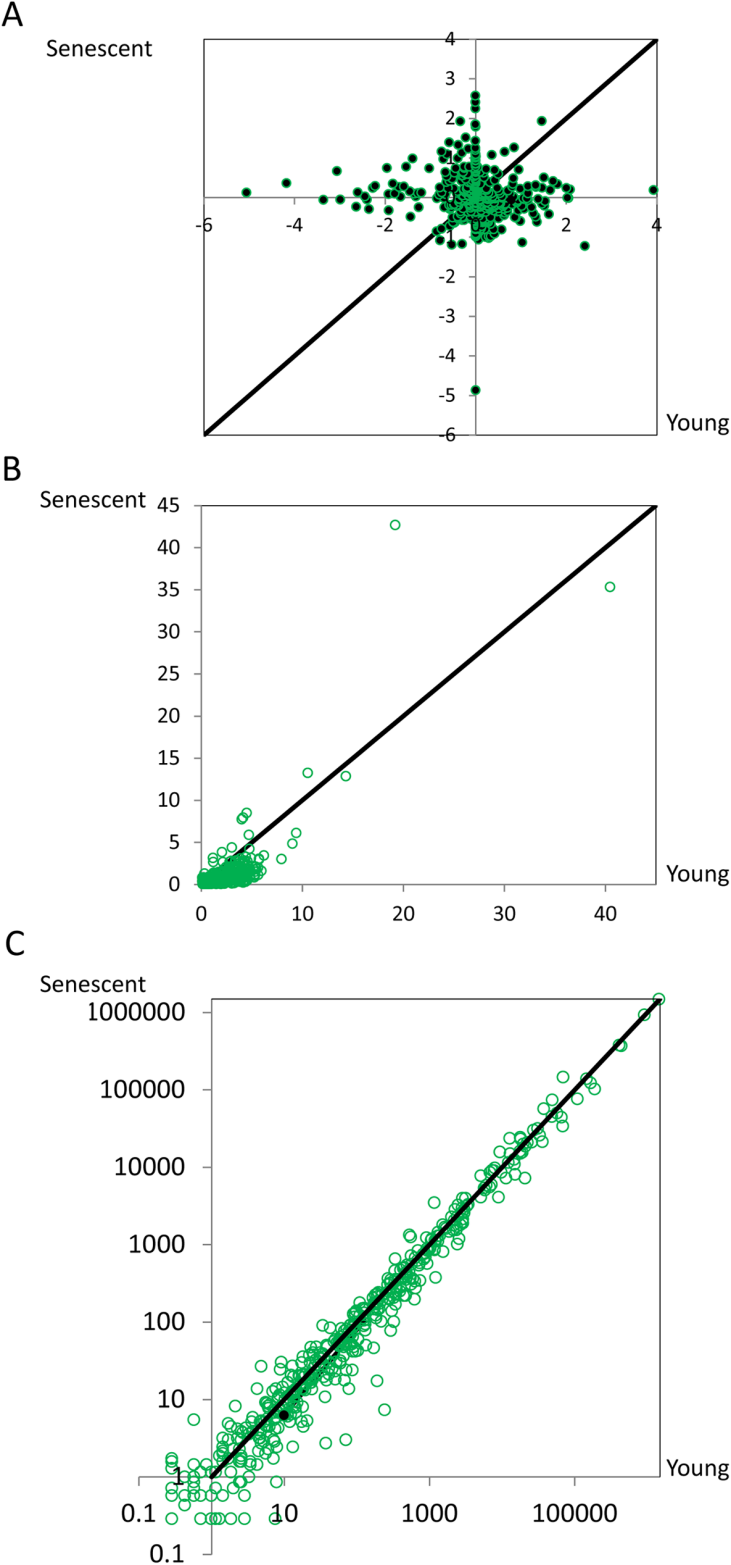
Comparative expression levels miRNAs in young cells (or tissues) (x axis) and senescent cells (or tissues) (y axis). (A) replicative senescence in HUVECs (the expression data was log2 transformed). (B) Oncogene-induced senescence in IMR90 cells. (C) Aging tissues in rhesus macaque brain.

To address the above questions, the global miRNAs expression level between senescent and young cell has been compared. By analyzing miRNAs expression data from normal growing and replicative senescent human umbilical vein endothelial cells (HUVECs) (GEO accession number: GSE37092), we found that senescent HUVECs show globally lower miRNAs expression level than young HUVECs (P = 3.328×10^−16^, Wilcoxon test, Fig 1A). It suggests that miRNA synthesis seems to be repressed but not activated in replicative senescent cells. Besides the replicative senescence type, we further analyzed miRNAs expression data (SupplData at http://www.cuilab.cn/) from oncogene H-Ras V12-induced senescent (RIS) IMR90 cells. Similar with replicative senescent cells, RIS senescent IMR90 cells also show globally lower miRNAs expression level than the untreated cells (P<2.2×10^−16^, Wilcoxon test, Fig 1B). The above results together suggest that miRNA synthesis seems to be attenuated in senescent cells. Because cells are the basic blocks of tissues and organisms, we then asked whether the above observation remains in aging tissues or organisms. For this purpose, we analyzed the miRNA expression data (GEO dataset: GSE18013) in young (developmental) and aging macaque brain. As expected, the rule keeps unchanged. The aging macaque brain showed globally lower miRNA expression profile than the young macaque brain (P<1.038×10^−10^, Wilcoxon test, Fig 1C), suggesting that globally miRNA is in low abundance in aging tissues as well.

To solid the above finding, we re-performed the above analysis for two more datasets, GSE64566 (epicardial adipose and subcutaneous adipose) and GSE37092 (human umbilical vein endothelial cell). For the GSE64566 dataset, we defined the samples older than 60 in age as the aging group and the other samples as the young group. As a result, the expression level of miRNAs in the young group is significantly higher than that in the aging group in both epicardial adipose (P = 0.01, Wilcoxon test) and subcutaneous adipose (P = 0.002, Wilcoxon test). For the human umbilical vein endothelial cell, the young cells have significantly higher miRNA expression level than the aging ones (P<2.2×10^−16^, Wilcoxon test).

### Oncogenic and tumor suppressing functions of changed miRNAs in aging cells or tissues

**Fig 2:**
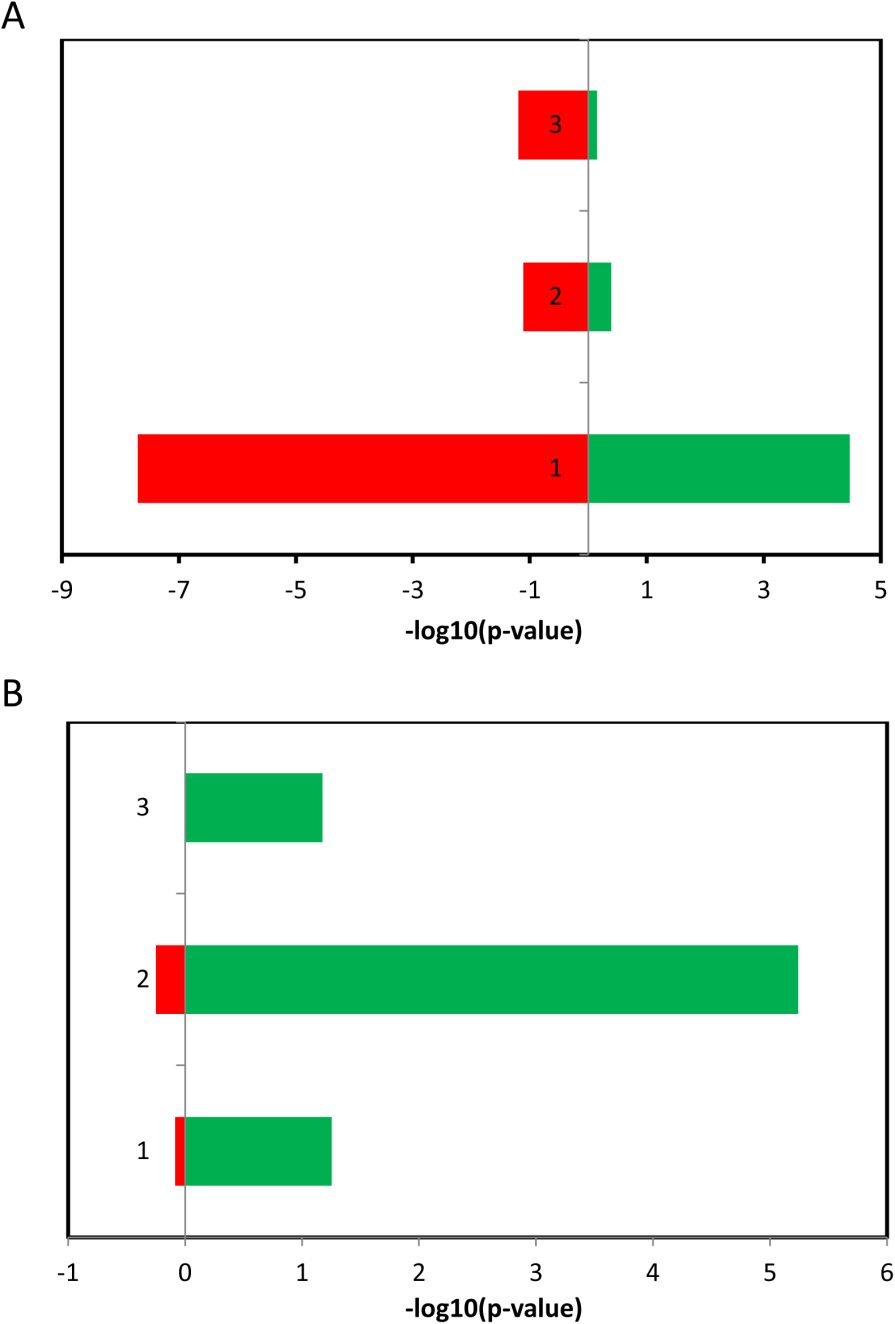
Result of enrichment analysis of deregulated miRNAs during cellular senescence in two cancer miRNA sets. (A) Tumor suppressing miRNAs. (B) oncogenic miRNAs. Red color represents senescent cells. Green color represents young cell. 1 Replicative senescence in HUVECs. 2 Oncogene-induced senescence in IMR90 cells. 3 Aging tissues in rhesus macaque brain.

As the intrinsic tumor-suppression mechanism, cellular senescence acts as a barrier that represses cell proliferation and the malignancy. Therefore, based on the analysis above, we are wondering the functions of miRNAs upregulated/downregulated in aging cells or tissues. It is known that miRNAs can be oncogenic or tumor suppressive [13]. Using TAM[12], the enrichment of deregulated miRNAs from the above three datasets was evaluated for both oncogenic and tumor suppressive miRNAs. As expected, for all three datasets, the miRNAs upregulated are more enriched in tumor suppressive miRNAs than those downregulated in aging cells and tissues (Fig 2A). In contrast, all three datasets showed opposite pattern in oncogenic miRNAs (Fig 2B). This result suggests that cellular senescence does exert tumor suppressing power accompanied with increased level of tumor suppressive miRNAs and antagonize oncogenic effect by decreasing the level of oncogenic miRNAs.

## Discussion

**Fig 3:**
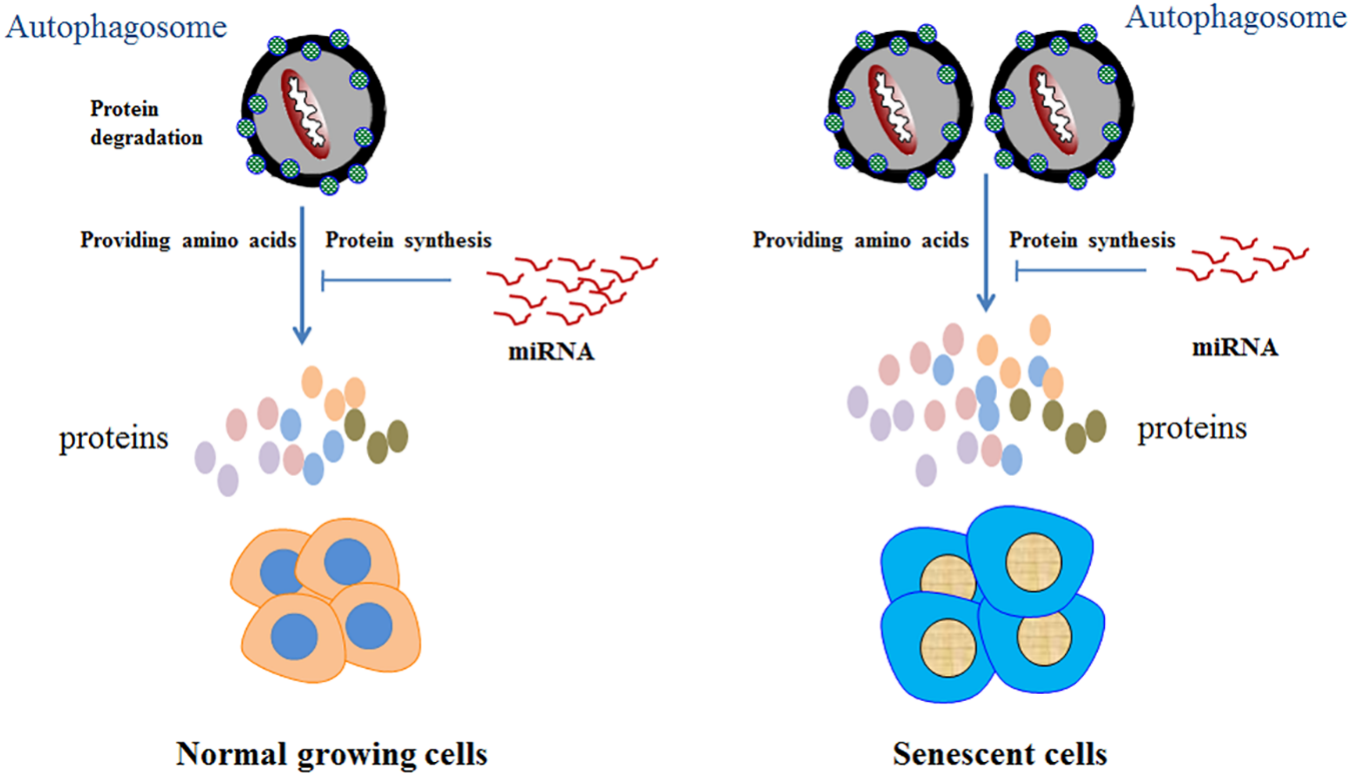
Model of relationship between miRNA abundance and protein accumulation during cellular senescence. Globally low expression of miRNAs in senescent cells decreased the suppression of RNA translating into proteins, which results in the accumulation of proteins accompanied with the increased proteins synthesis mediated by autophagy.

In summary, we revealed that global profile of miRNAs expression is decreased during cellular senescence. This indicates that miRNAs synthesis seems to be repressed but not activated during cellular senescence. Considering that the main function of miRNAs are to repress the translation of target mRNAs. This may present a new explanation to the phenomenon of protein accumulation during cellular senescence. That is, the global decreased miRNA level leads to the released repression on mRNAs, thus facilitates to increase the protein abundance. We summarized this hypothesis in Fig 3 which suggests that protein accumulation in senescent cells maight be also resulted from inhibited repression of mRNA translation by miRNAs but not only from activated protein synthesis triggered by authophagy. Although the exact mechanism linking miRNAs, cellular senescence, authophagy, and protein synthesis remains largely unknown, a possible role for miRNAs in cellular senescence at least presents a new opinion for the reason of protein accumulation in senescent cells.

However, it should be noted that the aging-associated miRNAs identified by transcriptomics is far from completeness. Therefore, developing bioinformatic methods to predict new aging-associated miRNAs would be important in the future. For doing so, algorithms in predicting miRNA-disease associations could be helpful [14–17].

## Conclusion

We revealed that globally the level of miRNAs is decreased during cellular senescence, which provided clues for the phenomenon of protein accumulation in aging cells.
